# Evaluating the Usability, Technical Performance, and Accuracy of Artificial Intelligence Scribes for Primary Care: Competitive Analysis

**DOI:** 10.2196/71434

**Published:** 2025-07-23

**Authors:** Emily Ha, Isabelle Choon-Kon-Yune, LaShawn Murray, Siying Luan, Enid Montague, Onil Bhattacharyya, Payal Agarwal

**Affiliations:** 1 Dalla Lana School of Public Health, University of Toronto, Toronto, ON, Canada; 2 Women's College Hospital Institute for Health System Solutions and Virtual Care, Women's College Hospital, 76 Grenville Street, 6th Floor, Toronto, ON, M5S 1B2, Canada, 1 4163236400; 3 Institute for Better Health, Trillium Health Partners, Mississauga, ON, Canada; 4 Department of Mechanical & Industrial Engineering, University of Toronto, Toronto, ON, Canada; 5 Institute for Health Policy, Management and Evaluation, University of Toronto, Toronto, ON, Canada; 6 Department of Family and Community Medicine, Temerty Faculty of Medicine, University of Toronto, Toronto, ON, Canada

**Keywords:** artificial intelligence, AI, AI scribes, clinical documentation, administrative burden, primary care, competitive analysis, evaluation

## Abstract

**Background:**

Primary care providers (PCPs) face significant burnout due to increasing administrative and documentation demands, contributing to job dissatisfaction and impacting care quality. Artificial intelligence (AI) scribes have emerged as potential solutions to reduce administrative burden by automating clinical documentation of patient encounters. Although AI scribes are gaining popularity in primary care, there is limited information on their usability, effectiveness, and accuracy.

**Objective:**

This study aimed to develop and apply an evaluation framework to systematically assess the usability, technical performance, and accuracy of various AI scribes used in primary care settings across Canada and the United States.

**Methods:**

We conducted a systematic comparison of a suite of AI scribes using competitive analysis methods. An evaluation framework was developed using expert usability approaches and human factors engineering principles and comprises 3 domains: usability, effectiveness and technical performance, and accuracy and quality. Audio files from 4 standardized patient encounters were used to generate transcripts and SOAP (Subjective, Objective, Assessment, and Plan)–format medical notes from each AI scribe. A verbatim transcript, detailed case notes, and physician-written medical notes for each audio file served as a benchmark for comparison against the AI-generated outputs. Applicable items were rated on a 3-point Likert scale (1=poor, 2=good, 3=excellent). Additional insights were gathered from clinical experts, vendor questionnaires, and public resources to support usability, effectiveness, and quality findings.

**Results:**

In total, 6 AI scribes were evaluated, with notable performance differences. Most AI scribes could be accessed via various platforms (n=4) and launched within common electronic medical records, though data exchange capabilities were limited. Nearly all AI scribes generated SOAP-format notes in approximately 1 minute for a 15-minute standardized encounter (n=5), though documentation time increased with encounter length and topic complexity. While all AI scribes produced good to excellent quality medical notes, none were consistently error-free. Common errors included deletion, omission, and SOAP structure errors. Factors such as extraneous conversations and multiple speakers impacted the accuracy of both the transcript and medical note, with some AI scribes producing excellent notes despite minor transcript issues and vice versa. Limitations in usability, technical performance, and accuracy suggest areas for improvement to fully realize AI scribes’ potential in reducing administrative burden for PCPs.

**Conclusions:**

This study offers one of the first systematic evaluations of the usability, effectiveness, and accuracy of a suite of AI scribes currently used in primary care, providing benchmark data for further research, policy, and practice. While AI scribes show promise in reducing documentation burdens, improvements and ongoing evaluations are essential to ensure safe and effective use. Future studies should assess AI scribe performance in real-world settings across diverse populations to support equitable and reliable applications.

## Introduction

### Overview

The majority of primary care providers (PCPs) in Canada and the United States report feelings of burnout, largely attributable to an increase in clinical documentation and administrative tasks [1,2], changes in practice patterns, and workforce shortages [3-5]. Since the widespread implementation of electronic medical records (EMRs) and evolving regulations and policies on medical documentation, PCPs, including family physicians and nurse practitioners, spend nearly half of their workday and an additional 40 hours per month completing administrative tasks after hours, such as clinical documentation of patient encounters and inbox management [6-10]. This increase in administrative burden has decreased provider satisfaction [2,10,11], impacted access to care as some providers have reduced clinical hours to manage administrative workload, and affected the patient-provider relationship [11,12]. Patient experience and satisfaction have also been impacted as PCPs have increasingly spent more time working in their EMR than on direct patient-facing care [12].

Advancements in artificial intelligence (AI) and machine learning have sparked interest in how automated processes can streamline and standardize clinical documentation in primary care. Several perspectives [13,14] and reviews [15,16] have described the need for AI scribes (also known as ambient scribes or digital scribes) to reduce administrative burden in primary care as well as other health care sectors. AI scribes use automatic speech recognition (ASR) and natural language processing (NLP) to record, transcribe, and automate clinical documentation. These tools are generally accessible via web browsers, mobile apps, or desktop applications, with some being integrated into EMRs while others function independently. The workflow typically involves clinicians activating the AI scribe during a patient encounter, where it listens and transcribes conversations and then generates a medical note thereafter. Some AI scribes also have capabilities to fill in billing codes or extract information to support diagnostic decision-making [17].

Several companies have developed AI scribes for use in primary care, promising to streamline workflows and reduce documentation time. However, there remain several concerns, including (1) the impact that AI scribes may have on the workflow of PCPs; (2) the effectiveness of these tools; and (3) the accuracy and quality of their outputs. To date, there are no evaluations that systematically compare the effectiveness of multiple AI scribes currently being used in primary care while also considering factors related to usability, technical performance, and accuracy [18-20]. In addition, given the risks primary care professionals and organizations may assume when adopting new technologies that potentially access, store, and retain personal health information (PHI), a thorough investigation of AI scribes currently being used in primary care is essential to ensure their safety and effectiveness.

### Objective

The study aimed to develop and apply an evaluation framework to systematically compare the usability, effectiveness, technical performance, and accuracy and quality of various AI scribes used in primary care settings in Canada and the United States.

## Methods

### Study Design

We conducted a systematic comparison of AI scribes using competitive analysis methods in the Virtual Care Lab at Women’s College Hospital (WCH) in Toronto, Ontario, Canada [21]. Competitive analysis methods are used to evaluate and compare the strengths, weaknesses, opportunities, and threats of competing products within a specific market to provide insights into market dynamics, identify gaps, and inform strategic decisions [22]. The analysis also incorporated expert usability approaches (eg, heuristic evaluation) and principles of human factors engineering [23-26]. Expert usability approaches enable the identification and examination of the current capabilities across several digital health tools to identify areas for improvement or features that are beneficial but may be missing [26]. Human factors engineering examines the interaction between systems, products, and their environment to enhance human performance, safety, and well-being [27]. Combining these methods enabled a comprehensive assessment and comparison of a suite of AI scribes while ensuring that the unique workflows of PCPs, their scope of work, and how they may be interacting with AI scribes within their clinical environments were considered.

The AI scribes included in this study were part of the Clinical Evaluation of Artificial Intelligence and Automation Technology to Reduce Administrative Burden in Primary Care project funded by the Ontario Ministry of Health through a Transfer Payment Agreement between Ontario Health and OntarioMD [28]. Only vendors who were compliant with Ontario’s Personal Health Information Protection Act (PHIPA) and adhered to best practices in data privacy and security were selected for participation in the clinical evaluation. These practices included supporting PCPs in ensuring that PHI was collected, used, and disclosed for authorized purposes only; supporting PCPs in securing valid consent for using PHI; implementing stringent safeguards against unauthorized access of PHI; and managing PHI retention and disposal appropriately. Vendors were also required to store and process data within Canada or, if stored outside, to provide adequate notice to health information custodians to support regulatory compliance and notification obligations.

### Evaluation Framework

#### Overview

Although there are instruments to assess physician note quality [29,30], a valid and reliable instrument that evaluates the usability, effectiveness, technical performance, and quality of the medical notes generated by AI does not exist. We developed an evaluation framework that adapted existing tools related to the usability (eg, user control, flexibility and efficiency of use, aesthetic and design, and help and documentation) [24] and provider documentation quality (eg, comprehensiveness, organization, conciseness, and usefulness) [29-31]. The framework was also informed by medical documentation policies and guidelines developed by governing and regulatory bodies in Canada, including the College of Physicians and Surgeons of Ontario (CPSO) [32] and the Canadian Medical Protective Association (CMPA) [33].

The evaluation framework has 12 items grouped into 3 domains: usability, effectiveness and technical performance, and accuracy and quality (Multimedia Appendix 1). The framework includes both quantitative and qualitative measures to capture various features and functions of AI scribes. For applicable items, a 3-point Likert scale (1=poor, 2=good, 3=excellent) was used to streamline the evaluation process. Definitions for each measure and level of the Likert scale were predefined to ensure consistent application of the evaluation framework.

#### Usability

Usability refers to the efficiency and satisfaction with which users can complete tasks on a given interface or platform [34]. Key usability factors for health care technologies in health care literature include user-software interaction, task alignment, and navigation [35]. Incorporating these factors, the usability domain in our evaluation framework assessed user interface, EMR compatibility and integration, and process flow of the AI scribes. Compatibility and integration measured the level of integration with common EMR systems in primary care in Ontario, focusing on efficient data exchange, interoperability, and standardized format adherence. User interface refers to the accessibility of each AI scribe across platforms and the graphical and interactive features users interact with. Process flow measured the steps (ie, mouse clicks and keystrokes) and time needed to sign in and launch the AI scribe. All measurements of time were conducted by team members in the same location with a consistent internet connection. Timing was averaged over 3 trials for each AI scribe to account for minor variability and excluded time spent entering credentials or textual information. Both user interface and EMR compatibility were evaluated using the 3-point Likert scale (with 1 representing poor usability or EMR compatibility and 3 representing excellent usability and EMR compatibility).

#### Effectiveness and Technical Performance

To assess effectiveness, average documentation time was measured as the time required to generate a complete medical note following a standardized 15-minute clinical encounter. This was calculated as the amount of time elapsed from the moment the recording stopped to the time in which a finalized medical note was generated by the AI scribe, excluding any additional edits or review by a PCP. For each AI scribe, documentation time was averaged across 12 appointments (4 standardized encounters, each repeated in 3 trials).

Technical performance refers to the ability of an AI scribe to generate reliable transcripts and medical notes when challenged with complicating factors, such as interruptions, loud background noise, or multiple speakers. Secondary audio files were used to introduce these elements during standardized patient-PCP interactions. Background noises included nonverbal sounds such as typing, paper shuffling, and ambient office sounds, as well as conversational noises from nearby voices. Interruptions and third-party speakers included brief interjections or conversations from other voices, mimicking real-world scenarios where individuals may briefly speak or disrupt the main patient-PCP interaction or if a caregiver is present. Both the transcripts and medical notes generated by each of the AI scribes were evaluated against the 3-point Likert scale (1=poor performance, 2=good performance, 3=excellent performance). “Excellent” performance was defined as accurately attributing statements to the correct speaker, maintaining the logical flow of conversation, and minimizing omissions or distortions. “Good” performance allowed for minor attribution errors or brief lapses that did not meaningfully impact the clinical content. “Poor” performance was characterized by major attribution errors, significant omissions, disorganized conversation flow, or outputs that impaired the clinical utility of the transcript or note.

#### Accuracy and Quality in Documentation

The accuracy and quality of the medical notes generated by the AI scribes were assessed across 7 items: accuracy, comprehensiveness, care plan, organization, comprehension, conciseness, and usefulness (Textbox 1). These items were informed by medical documentation policies and guidelines developed by the CPSO [32] and the CMPA [33], validated quality frameworks that assessed the accuracy and quality of medical notes written by physicians and medical scribes [30,36], and early evaluations on the accuracy of AI scribes in other health care settings [31]. Each item was measured against a 3-point Likert scale and scores were averaged to determine a single performance score (with 1 representing poor accuracy and quality and 3 representing excellent accuracy and quality). The transcript was also assessed for overall accuracy and quality.

Textbox 1. **Description of items to evaluate the accuracy and quality of medical notes generated by an artificial intelligence (AI) scribe.**

**Accuracy**

The medical note contains information that is true and free from errors or hallucinations.

**Comprehensiveness**

The medical note includes complete documentation of all relevant patient information, including medical history, examination findings, diagnostic results, and treatment plans without omissions.

**Care Plan**

The medical note provides a holistic understanding of the patient’s health status. The medical note allows health care professionals to readily interpret the patient’s health status and develop a plan of care.

**Organization**

The medical note is well-structured, adhering to the SOAP (Subjective, Objective, Assessment, and Plan) format.

**Comprehension**

The medical note is accessible and devoid of ambiguity or difficult-to-understand terms, phrases, or sections. The medical note allows health care professionals to readily interpret the patient’s clinical status and make informed decisions.

**Conciseness**

The medical note succinctly and effectively conveys all essential information, avoiding unnecessary elaboration or redundancy.

**Usefulness**

The medical note presents pertinent clinical information in a clear, concise, and actionable manner, facilitating effective communication, decision-making, and continuity of care among health care professionals involved in the patient’s care.

We also conducted an error analysis to identify the types of mistakes made by the AI scribes when generating transcripts and medical notes using error classification schemes developed for ASR systems [37-39]. Error types included deletion or omission errors and errors in names, proper nouns, numbers, punctuation, medication names, medical terminology, homonyms, and the Subjective, Objective, Assessment, and Plan (SOAP) format.

### Applying the Evaluation Framework

To conduct the competitive analysis, 4 research team members (EH, IC-K-Y, LM, and SL) had an “unlimited” or “premium” license for each AI scribe. Each team member played the audio files from 4 different simulated clinical encounters between a PCP and a standardized patient to generate transcripts and medical notes in the SOAP format from each AI scribe. The audio files were obtained from the College of Family Physicians of Canada Certification Examination in Family Medicine website and were selected because they portrayed common clinical encounters and patient populations observed in primary care. Each audio file also had a verbatim transcript, detailed case notes, and SOAP-format notes written by PCPs, all of which served as a rubric for comparing to the AI scribe-generated outputs. The use of audio files helped minimize discrepancies and challenges related to variations in clinical presentations; thus, providing uniform encounters to each AI scribe and allowing researchers to verify results against the original source for more accurate assessments. The SOAP format was selected for documentation as it was a universally available template across all AI scribe products and is one of the most recommended and widely used methods for documenting a patient encounter in primary care [32].

Team members individually evaluated items in the framework, collaborating weekly to resolve any rating discrepancies through discussion and consensus. If an agreement could not be reached, a fifth team member adjudicated. Clinical experts and family physicians also reviewed each measure, adding insights on usability, effectiveness, technical performance, and output accuracy and quality for each AI scribe. Their feedback was incorporated through a second round of review and consensus meetings among the core evaluation team. In addition, data from a questionnaire completed by the AI scribe vendors about their products’ capabilities was analyzed, and publicly available resources, including company websites, product documentation, user manuals, whitepapers, case studies, and user testimonials were reviewed.

### Ethical Considerations

The study was formally reviewed by institutional authorities at WCH and received research ethics approval from the WCH Assessment Process for Quality Improvement Projects pathway (APQIP #2023‐0059). The study was conducted within a controlled simulation environment using audio recordings of standardized clinical encounters between a PCP and a standardized patient. The audio recordings are publicly available to use for educational and research purposes. Results generated as part of this study were based solely on these standardized patient encounters and did not include human participants or the collection of any PHI. All research data were securely stored on password-protected internal servers with access restricted to authorized study personnel.

## Results

### Overview

A total of 6 AI scribes were selected for evaluation based on their market availability and compliance with data privacy and security regulations in Ontario (ie, PHIPA). To highlight the general capabilities of AI scribes, specific product names are not used; however, when referring to a particular AI scribe product, they are labeled as AI scribes #1 through #6. Table 1 provides an overview of the health care sectors and users for each of the AI scribes. While 2 AI scribes were exclusively used in primary care (AI scribes #1 and #4), the remaining 4 were also deployed in other health care sectors, including acute care, home care, palliative care, and community services. Of these 4 AI scribes, 3 were used by a broader range of health care professionals, including nurses, pharmacists, dentists, veterinarians, and administrative staff, in addition to physicians. The number of active, paid subscribers for each AI scribe varied significantly, from 20 for AI scribe #4 to over 21,000 users for AI scribe #6. Half of the AI scribes supported languages other than English (AI scribes #2, #3, and #5), while the remaining 3 only supported English but reported that they were developing additional language options (eg, French and Spanish). The performance of these AI scribes in non-English languages was not evaluated in this analysis.

**Table 1.** Overview of supported health care settings and users across different artificial intelligence (AI) scribe products.

Context	AI scribes					
	AI scribe #1	AI scribe #2	AI scribe #3	AI scribe #4	AI scribe #5	AI scribe #6
Care settings^a^	Primary care	Primary care, acute and ambulatory care, social and community services, home care, palliative care, mental health and psychiatry, pediatrics, rehabilitation services, dental care, optometry, surgery, and veterinary medicine	Primary care, acute and ambulatory care, palliative care, and home care	Primary care	Primary care, acute and ambulatory care, community services, and mental health and psychiatry	Primary care and acute and ambulatory care
Main users^b^	Physicians	Physicians, nurses, other regulated health care professionals^c^, and administrative staff	Physicians, nurses, other regulated health care professionals^c^, and administrative staff	Physicians	Physicians, nurses, and other regulated health care professionals^c^	Physicians
Number of users^d^	100	13,000	360	20	4000	21,000
Supported languages	English	English, French, and 50 other languages	English, French, and 20 other languages	English	English and French	English

a All health care sectors that the AI scribe was used in.

b Types of clinicians and health care professionals that the AI scribe was marketed to, and who had an active, paid subscription to use the AI scribe (ie, users).

c Regulated health professionals include pharmacists, dentists, occupational therapists, physiotherapists, social workers, psychologists, and therapists.

d Approximate number of current users with an active, paid subscription for the AI scribe.

### Usability

Table 2 presents the results of the usability assessment. Of the 6 AI scribes evaluated, 4 were accessible on multiple platforms (desktop or laptop and mobile or tablet devices); thus, receiving an excellent form factor rating. In contrast, AI scribes #4 and #6 could only be accessed on a single platform, potentially limiting accessibility in diverse clinical settings. Four of the AI scribes also demonstrated some level of EMR integration. AI scribes #1 and #3 exhibited the highest degree of integration as they were compatible with multiple EMRs used in primary care in Canada and could exchange metadata (eg, schedules, visit details, previous notes, and summaries) and patient information (eg, name, date of birth, and health card number) when launched from within the EMR. AI scribe #5 could be launched within a single EMR, but no data exchange was available. AI scribe #6 did not connect directly to common EMRs in primary care but allowed users to transfer notes through a separate application. To add the medical note to the EMR, only AI scribe #5 offered a seamless drag-and-drop note transfer into the EMR with a single mouse click. For AI scribes #1‐#4, users would have to toggle between the AI scribe and EMR interfaces to manually copy-paste notes, adding to the number of mouse clicks or keystrokes required to use the AI scribe. AI scribe #6 used a separate program to transfer the transcript and medical note from the mobile app to the EMR.

**Table 2.** Comparison of usability features across different artificial intelligence (AI) scribe products.

Usability measures	AI scribes					
	AI scribe #1	AI scribe #2	AI scribe #3	AI scribe #4	AI scribe #5	AI scribe #6
User interface						
Main platforms used to access the AI scribe^a^	Supported on Google Chrome, Mozilla Firefox, Apple Safari, and Microsoft Edge	Supported on all web browsers	Supported on Google Chrome, Mozilla Firefox, and Microsoft Edge	Desktop application	Google Chrome extension but also available on other Web browsers, although not formally supported	Mobile app
Supported mobile and tablet devices	Mobile-friendly via phone’s web browser	Mobile-friendly via phone’s web browser	Mobile-friendly via phone’s web browser	N/A	Native application for iOS and Android devices	Native application available for iOS and Android devices
Form factor^b^	Excellent	Excellent	Excellent	Poor	Excellent	Poor
EMR integration						
EMR integration	Excellent	Poor	Excellent	Poor	Excellent	Good
Ability to transfer medical note	Good	Good	Good	Good	Excellent	Poor
Process flow						
Steps to sign-in and launch the AI scribe	5	7	6	6	4	4
Average time to sign-in and launch the AI scribe^c^	10.01 sec^d^	13.58 sec	26.36 sec	14.59 sec	11.63 sec	7.21 sec
Factor authentication (FA)	1FA^e^	1FA	2FA^f^	1FA	2FA	1FA
Ease of Restarting	Good	Good	Good	Good	Good	Good

a Medium or technology through which users can access and interact with the AI scribe, considering the various types of interfaces and devices that enable user access.

b Design and accessibility of platforms required to use the AI scribe, particularly in relation to its ease of access in primary care settings.

c Approximate amount of time needed to sign in and launch the AI scribe for use during a patient encounter. This measure was averaged across 3 runs and did not include time spent typing when entering credentials or textual information.

d sec: seconds.

e 1FA: single-factor authentication.

f 2FA: 2-factor authentication.

Other processes affecting usability, such as sign-in and launch, varied across products. All AI scribes required user authentication for sign-in, with 4 AI scribes using single-factor authentication and 2 implementing 2-factor authentication. There did not appear to be a link between the number of steps and the amount of time it took to sign in and launch the AI scribe. The encounter initiation processes also varied across AI scribes; some required preloading appointment data (eg, patient name and appointment time), while others could start recording immediately. Each AI scribe featured a visible “start encounter” button, and all except AI scribe #4 provided live transcription. Transitioning between patient encounters required users to save the medical note before moving on to the next patient.

### Effectiveness and Technical Performance

On average, for a 15-minute clinical encounter between a patient and PCP, nearly all AI scribes generated a SOAP-format medical note within 1 minute of pressing the stop recording button, with AI scribe #4 taking almost twice as long (Table 3). Documentation time for all AI scribes was affected by the encounter length and the complexity of the topics discussed. Encounters involving multiple or complex topics, or extended, nonlinear conversations—including rapport-building exchanges not directly related to medical care—generally increased the documentation time of the AI scribes.

**Table 3.** Effectiveness and technical performance across different artificial intelligence (AI) scribe products.

Performance measures	AI scribes					
	AI scribe #1	AI scribe #2	AI scribe #3	AI scribe #4	AI scribe #5	AI scribe #6
Effectiveness						
Average documentation time	41.87 sec^a^	53.54 sec	42.8 sec	1:40 min^b^	32.4 sec	20.66 sec
Technical performance						
Background noise	Excellent	Excellent	Excellent	Excellent	Excellent	Excellent
Interruptions	Excellent	Good	Excellent	Good	Good	Poor
Multiple speakers	Good	Poor	Good	Good	Poor	Good

a sec: seconds.

b min: minutes.

The performance of the AI scribes also varied when faced with complicating factors. All AI scribes successfully omitted nonconversational background noises. In contrast, only AI scribe #1 effectively filtered out interruptions and extraneous conversations, ensuring only the patient-PCP dialogue appeared in the transcript and medical note. AI scribes #2‐#5 included brief phrases from interruptions or extraneous conversations in the transcript but excluded them from the medical note. AI scribe #6, however, mistakenly incorporated information from the interruption into both the transcript and the medical note.

The ability of the AI scribes to manage conversations among 3 or more speakers also varied. AI scribes #1, #3, #4, and #6 received a “good” rating, effectively distinguishing between speakers and accurately assigning dialogue to speakers in the transcripts. In contrast, AI scribes #2 and #5 received a “poor” rating, often failing to differentiate between more than 2 speakers when generating the transcript. These errors led to inaccuracies in the medical note, where statements made by a third speaker were incorrectly attributed to the patient.

### Accuracy and Quality in Documentation

Table 4 summarizes the evaluation findings on documentation accuracy and quality. None of the AI scribes consistently produced fully accurate and error-free transcripts or medical notes, although the types of errors varied. While most patient-PCP conversations were transcribed accurately, frequent grammatical and syntactical errors, such as repeated or omitted words, were observed, particularly among AI scribes #1 and #3‐#5. These AI scribes received an overall rating of “good” for transcript accuracy and quality, as these issues could affect the transcripts’ usefulness, making it harder for users to reference them quickly during later reviews, although it did not affect overall content.

**Table 4.** Evaluation of the accuracy and quality of the transcript and medical note generated by the artificial intelligence (AI) scribe.

Accuracy measures	AI scribe					
	AI scribe #1	AI scribe #2	AI scribe #3	AI scribe #4	AI scribe #5	AI scribe #6
Accuracy and quality						
Transcript	Good	Excellent	Good	Good	Good	Excellent
Medical note	Excellent	Excellent	Excellent	Good	Good	Good
Error analysis^a^^,^^b^						
Deleted words or omissions^c^	✓	✓	✓	✓	✓	✓
Generalizations^d^				✓	✓	✓
SOAP^e^ structure^f^		✓	✓	✓		

a Checkmarks indicate that at least one occurrence of the error was observed.

b Error types not observed include errors in names, proper nouns, numbers, punctuation, medication names, or homonyms.

c Error where essential information is left out or not included, which can lead to incomplete understanding or documentation of the intended content or context.

d Error where specific information is simplified or replaced with a broader, less precise term, potentially leading to a loss of detail or nuance that could affect the accuracy or clarity of the information.

e SOAP: Subjective, Objective, Assessment, or Plan.

f Documentation error where information is incorrectly categorized within the SOAP sections of a medical note, potentially leading to confusion or misinterpretation of the clinical details.

For medical note accuracy and quality, 3 AI scribes (#4‐#6) produced notes rated as “good” quality, while the remaining 3 (AI scribes #1‐#3) produced “excellent” quality notes. An “excellent” rating indicated strong relative performance among the evaluated AI scribes rather than complete accuracy. The “excellent” notes were comprehensive, well-organized, and free from hallucinations or contradictory information, providing sufficient detail to accurately reflect the patient’s clinical status and support informed decision-making. Interestingly, AI scribes that generated good-quality transcripts were still capable of producing excellent-quality medical notes (AI scribes #1 and #3), and the reverse was also true (AI scribe #6).

In the error analysis, errors involving names, proper nouns, numbers, punctuation, medication names, or homonyms were not observed. However, deletion or omission errors were observed across all AI scribes. The most frequent omissions involved details about the history of the presenting illness, social history, or lifestyle factors, with the extent of omissions varying across AI scribes. For example, in one clinical scenario, AI scribes #2, #3, and #4 omitted dietary information relevant to the encounter but still received a good rating, as this information could have been documented elsewhere in the patient’s chart. Conversely, AI scribe #6 consistently omitted new social history details directly relevant to the presenting illness. AI scribes #4‐#6 also made generalization errors, simplifying medical terms in ways that lost specificity or nuance, such as reducing “Colles fracture” to “fracture” or even “dog fracture.” In addition, AI scribes #2‐#4 displayed SOAP structure errors, such as misclassifying information in the Objective section despite the absence of a physical examination.

## Discussion

### Principal Findings

We conducted one of the first studies, to our knowledge, that systematically evaluated the usability, effectiveness, technical performance, and accuracy of 6 AI scribes developed for or used in primary care settings in Canada, including some tools that are also used in the United States. By leveraging competitive analysis methods, expert usability approaches, and human factors engineering, we developed a comprehensive evaluation framework that revealed a rapidly evolving AI scribe market and notable differences in performance across AI scribes.

Of the AI scribes included in this evaluation, most were accessible across multiple platforms, including desktops, laptops, mobile devices, and tablets, and could be launched directly within the EMR for easier access, although data exchange was minimal. In general, nearly all AI scribes generated a SOAP-format medical note in approximately 1 minute for a standardized 15-minute encounter, although documentation length increased based on the length of the encounter and complexity of topics discussed. While all AI scribes produced good to excellent quality medical notes, persistent deletion and omission errors highlighted the continued need for review and editing by PCPs. In addition, several factors were found to affect the quality and accuracy of both transcripts and medical notes, including extraneous conversations, encounters with multiple speakers, and the complexity of conditions discussed. Interestingly, AI scribes that produced good-quality transcripts could generate excellent-quality medical notes, and conversely, transcripts of excellent quality did not always result in equally high-quality medical notes. Despite these promising capabilities, limitations related to usability, technical performance, and accuracy remain that must be addressed to ensure AI scribes continue to reduce administrative burdens effectively.

### Comparison With Other Work

While studies on the usability of AI scribes in primary care remain limited, our findings are consistent with broader research on the usability of digital health technologies. For example, inadequate access, poor usability of platforms, and lack of workflow integration are known factors that contribute to PCP burnout, whereas enhanced accessibility and robust EMR integration are associated with improved PCP well-being and productivity [40,41]. As demonstrated in this study, flexible access to AI scribes across multiple platforms—such as mobile devices, tablets, and desktops—may enhance their usability by aligning better with PCPs’ varied workflows and preferences. In addition, as highlighted in several commentaries, AI scribes remain an unregulated digital health tool [42,43]. While the current level of EMR integration may be sufficient in the absence of regulation, data privacy and security laws, like those in Ontario, Canada, place the responsibility on PCPs and institutions to understand privacy implications, assess risks, and ensure that AI scribe software functions as expected [42,43]. These considerations underscore the need for regulatory guidance and transparency in the development of AI scribe technology to support usability, enhance EMR integration, and maintain patient safety in primary care settings.

Studies on the accuracy and quality of transcripts and medical notes produced by AI scribes are emerging. For instance, Tierney et al [31] used a modified 9-item Physician Documentation Quality Instrument (PDQI-9) to evaluate the quality of notes produced by an AI scribe used in primary care, pediatric, hospital, mental health, surgical, and emergency care settings. Their regional pilot demonstrated that while the AI scribe produced high-quality notes, minor hallucinations and omission errors were common—similar to the error types observed in our study. In addition, a study on a commercially available Dutch AI scribe found that fully automated notes, without clinician editing, had poorer PDQI-9 scores, higher word counts, and reduced lexical diversity compared to those reviewed by clinicians [44]. Our evaluation of 6 AI scribes expanded upon this work and demonstrated that various AI scribes can produce good to excellent quality notes, though none were entirely error-free and likely require some editing by users. Compared with the PDQI-9 and other tools used to evaluate note quality, our framework consists of 7 items that focus on characteristics unique to AI-generated outputs, such as hallucinations, redundancy, and bias. Our framework also considers the ability of AI-generated notes to capture relevant patient information in a concise and actionable manner, with a particular emphasis on attributes like the Care Plan. It is designed to be suitable for SOAP-format medical notes, though it can be adapted to other formats, highlighting both similarities and differences in focus and scope.

Despite the promise of AI scribes, recent evaluations of large language models (LLMs), such as GPT-3.5 and Open AI’s ChatGPT-4, urge caution before integrating AI-powered tools into clinical documentation workflows. In these studies, LLMs were fed medical evidence or transcripts of audio-recorded patient-provider conversations to generate summaries [45,46]. Findings revealed that LLMs frequently produce factually inconsistent summaries, with error types varying across replicates of the same case [45]. Performance also differed significantly across different LLMs [46]. In contrast to general purpose LLMs, such as ChatGPT, the AI scribes evaluated in our study primarily relied on conventional pipelines combining ASR systems and NLP models that were specifically fine-tuned for clinical documentation tasks. While some AI scribe vendors may be beginning to integrate LLM-based approaches, most tools included in this evaluation were optimized separately for ASR and NLP functions. Notably, the distinct functions of ASR systems and NLP within AI scribes likely explain why ‘excellent’ transcripts may sometimes accompany ‘good’ medical notes, or vice versa, as observed in our study. ASR systems are optimized to capture spoken words accurately, while NLP focuses on summarizing, organizing, and emphasizing clinically relevant information [14]. Consequently, some AI scribes may excel in extracting key details for notes despite minor transcript inaccuracies, while others may produce precise transcripts but lack effective summarization. A key takeaway is that the performance of AI scribes is expected to vary depending on the specific ASR and NLP systems used, and performance will likely change over time as these models are retrained on new data. The variability in performance within and across AI scribes underscores the need for ongoing evaluation and oversight to ensure that these tools continue to meet clinical documentation needs reliably [47]. Collectively, these findings highlight the importance of PCPs reviewing and editing AI-generated medical notes to ensure documentation accuracy and quality.

Overall, our study has important implications for research, policy, and practice. For research, the findings highlight the need for further large-scale studies to evaluate the usability, accuracy, and long-term impact of AI scribes, especially in real-world clinical environments. This research also raises important equity considerations, as the performance of various AI scribes may differ across PCP and patient populations or when used to support languages other than English [46,48]. For policy and practice, our findings provide benchmark data on the current performance of 6 AI scribes used in primary care settings, guiding users, institutions, and other stakeholders on both the strengths and areas needing improvement. This work can also set the foundation for broader implementation strategies that balance innovation with safety, equity, and accountability in digital health technology. Furthermore, there is a critical need for the ongoing evaluation of AI scribes and other AI tools in a manner that is both timely and rigorous. With the rapid evolution of AI technology, continuous assessment is necessary to ensure that these tools remain relevant, effective, and safe for diverse clinical contexts. Evaluations must be methodologically robust to address potential biases, assess long-term impacts, and ensure that the tools meet the needs of all patient and provider populations equitably to build trust, maintain accountability, and maximize the transformative potential of AI in health care.

### Limitations

There are limitations to consider when interpreting findings. First, the evaluation was conducted in a controlled laboratory setting, which may not fully capture the nuances and complexities of real-world clinical environments. While we used standardized audio files representing typical primary care encounters, these simulations cannot entirely replicate live, unscripted interactions. It is also possible that some AI scribes were partially trained or fine-tuned using publicly available clinical audio datasets, which could introduce overlap with the types of audio files used in our evaluation. Although we cannot confirm the specific training sources used by each vendor (as this information was often proprietary), we acknowledge this as a limitation and recognize that model performance may differ when evaluated using de novo, real-world clinical encounter data. Second, we evaluated 6 AI scribes, although there are a countless number of AI scribes available globally. While the selected vendors reflect those used in primary care settings in Canada and the United States, the results may not capture the full range of features offered by all AI scribes. In addition, AI scribes and automation technologies are rapidly evolving. Although the evaluation framework may be applied to continuously evaluate AI scribes, as the underlying ASR and NLP models are retrained, findings represent the relative performance of each AI scribe at a single point in time and may not reflect any subsequent versions released since the time of the study. Finally, we assessed documentation quality using the SOAP format to ensure consistent evaluation of outputs across all AI scribes that were evaluated. While this may not represent the variety of templates or the specialized expertise required to select the most appropriate template for different clinical scenarios, the SOAP format remains the most widely recommended method for documenting clinical encounters in primary care, and its use in this study provided a consistent basis for comparison.

### Conclusion

In conclusion, our findings demonstrate that developing a comprehensive framework to evaluate AI scribes is feasible, and findings from the competitive analysis underscore the value of AI scribes as a promising tool to alleviate administrative burden in primary care. However, findings also emphasize the need for ongoing enhancements to improve the usability, technical performance, and accuracy of AI scribes. Although AI scribes can generate good to excellent quality medical notes, user editing and review remain essential. Greater EMR integration and personalized customization are also needed to align with the varied documentation practices of PCPs. Overall, ongoing evaluation is crucial to ensure these technologies effectively support PCPs while enhancing safety, accuracy, and efficiency for both providers and patients. Future research should also explore AI scribe performance in dynamic, real-world settings.

## Supplementary material

10.2196/71434 Multimedia Appendix 1 Overview of evaluation framework domains, items, and assessment method.

## Abbreviations

AI: artificial intelligence
APQIP: Assessment Process for Quality Improvement Projects
ASR: automatic speech recognition
CMPA: Canadian Medical Protective Agency
CPSO: College of Physicians and Surgeons of Ontario
EMR: electronic medical record
LMM: large language model
NLP: natural language processing
PCP: primary care provider
PDQI-9: 9-item Physician Documentation Quality Instrument
PHI: personal health information
PHIPA: Personal Health Information Protection Act
SOAP: Subjective, Objective, Assessment, and Plan
WCH: Women’s College Hospital
