# Supplementary material for: Evaluating the Usability, Technical Performance, and Accuracy of Artificial Intelligence Scribes for Primary Care: Competitive Analysis
Source: JMIR Hum Factors. 2025 Jul 23;12:e71434. doi: 10.2196/71434 (PMC12309782; doi:10.2196/71434)
Supplement: Multimedia Appendix 1 [file humanfactors-v12-e71434-s001.docx]

Multimedia Appendix 1

**Table S1.** Overview of evaluation framework domains, items, and assessment method

| **Domain** | **Item** | **Subitems** | **Description** | **Measure Type** | **Assessment Method** |
| --- | --- | --- | --- | --- | --- |
| **Usability** | User interface | Main platform(s) used to access the artificial intelligence (AI) scribe | Type of platform(s) or interface(s) through which users’ access and interact with the AI scribe. | Qualitative | Descriptive |
|  |  | Supported mobile and tablet devices | Availability and functionality of the AI scribe on mobile and tablet devices (eg, iOS, Android). | Qualitative | Descriptive |
|  |  | Form factor | Design, accessibility, and physical setup requirements of the AI scribe platform, particularly for use in primary care settings. | Quantitative  (3-point Likert) | 1 = Poor,  2 = Good,  3 = Excellent |
|  | Electronic medical record (EMR) compatibility and integration | EMR integration | Level of integration with common EMR systems used in primary care in Ontario, focusing on efficient data exchange, interoperability, and format adherence. | Quantitative  (3-point Likert) | 1 = Poor,  2 = Good,  3 = Excellent |
|  |  | Ability to transfer medical note | Ability of the AI scribe and/or user to directly transfer generated medical notes to the EMR. | Quantitative  (3-point Likert) | 1 = Poor,  2 = Good,  3 = Excellent |
|  | Process flow | Steps to sign-in and launch the AI scribe | Number of mouse clicks and keystrokes required to sign in and launch the AI scribe. | Quantitative | Count of steps |
|  |  | Average time to sign-in and launch the AI scribe | Average time, measured over 3 trials, required to sign in and launch the AI scribe, excluding time spent entering credentials or textual information. | Quantitative | Average time in seconds |
|  |  | Factor authentication (FA) | Use and type of multifactor authentication (eg, SMS, authenticator apps) required to access the AI scribe platform. | Qualitative | Descriptive |
|  |  | Ease of restarting | Ability to efficiently save a completed note and transition to documentation for a new patient encounter without disruption. | Quantitative  (3-point Likert) | 1 = Poor,  2 = Good,  3 = Excellent |
| **Effectiveness and Technical Performance** | Documentation time | Average documentation time | Average time elapsed between the end of an encounter recording and the generation of a complete medical note (excluding user edits). | Quantitative | Average time in minutes |
|  | Complicating Factors | Handling of background noise | Ability to maintain transcript and note quality in the presence of nonverbal sounds (eg, typing, shuffling, ambient noise). | Quantitative  (3-point Likert) | 1 = Poor,  2 = Good,  3 = Excellent |
|  |  | Handling of interruptions | Ability to preserve conversation flow and documentation when disrupted by third-party interruptions. | Quantitative  (3-point Likert) | 1 = Poor,  2 = Good,  3 = Excellent |
|  |  | Handling of multiple speakers | Ability to distinguish and document multiple speakers accurately during a clinical encounter. | Quantitative  (3-point Likert) | 1 = Poor,  2 = Good,  3 = Excellent |
| **Accuracy and Quality in Documentation** | Accuracy | The medical note contains information that is true and free from errors or hallucinations. | | Quantitative  (3-point Likert) | 1 = Poor,  2 = Good,  3 = Excellent |
|  | Comprehensiveness | The medical note includes complete documentation of all relevant patient information, including medical history, examination findings, diagnostic results, and treatment plans without omissions. | | Quantitative  (3-point Likert) | 1 = Poor,  2 = Good,  3 = Excellent |
|  | Care Plan | The medical note provides a holistic understanding of the patient’s health status. The medical note allows health care professionals to readily interpret the patient’s health status and develop a plan of care. | | Quantitative  (3-point Likert) | 1 = Poor,  2 = Good,  3 = Excellent |
|  | Organization | The medical note is well-structured, adhering to the SOAP format (Subjective, Objective, Assessment, and Plan). | | Quantitative  (3-point Likert) | 1 = Poor,  2 = Good,  3 = Excellent |
|  | Comprehension | The medical note is accessible and devoid from ambiguity or difficult-to-understand terms, phrases, or sections. The medical note allows healthcare professionals to readily interpret the patient’s clinical status and make informed decisions. | | Quantitative  (3-point Likert) | 1 = Poor,  2 = Good,  3 = Excellent |
|  | Conciseness | The medical note succinctly and effectively conveys all essential information, avoiding unnecessary elaboration or redundancy. | | Quantitative  (3-point Likert) | 1 = Poor,  2 = Good,  3 = Excellent |
|  | Usefulness | The medical note presents pertinent clinical information in a clear, concise, and actionable manner, facilitating effective communication, decision-making, and continuity of care among health care professionals involved in the patient’s care. | | Quantitative  (3-point Likert) | 1 = Poor,  2 = Good,  3 = Excellent |
